# Representation of Women Among Grand Rounds Speakers at an Academic Health System Early in the COVID-19 Pandemic

**DOI:** 10.7759/cureus.41150

**Published:** 2023-06-29

**Authors:** Prarthna V Bhardwaj, Anna C Clarke, Alexander Knee, Amy S Gottlieb, Kathryn A Jobbins

**Affiliations:** 1 Hematology Oncology, University of Massachusetts Chan Medical School - Baystate, Springfield, USA; 2 Internal Medicine, University of Massachusetts Chan Medical School - Baystate, Springfield, USA; 3 Epidemiology and Biostatistics Research Core, Office of Research, Baystate Medical Center, Springfield, USA; 4 Office of Faculty Affairs, University of Massachusetts Chan Medical School - Baystate, Springfield, USA

**Keywords:** female speakers, women in medicine, covid-19, women speakers, grand rounds, academic promotion, gender representation

## Abstract

Introduction

An invitation to speak at grand rounds (GR) is considered an honor and an activity important for academic promotion. The aim of this study was to determine the representation of women among invited speakers at departmental GR and assess the impact of the COVID-19 pandemic on it.

Methods

We conducted a retrospective descriptive study on gender differences in all GR speakers between January 2019 and June 2021 at an academic health system in Western Massachusetts. We calculated the overall percentage of women presenters and compared it with the percentage of women faculty at our institution and nationally. To evaluate the impact of COVID-19 on this association, we calculated the absolute percentage difference between women and men speakers using the date of March 13, 2020, as the cut-off and conducted a sensitivity analysis using June 15, 2020, as the cut-off.

Results

During the study period, 46% (276/607) of GR speakers at our institution were women. This percentage reflected the percentage of the women faculty overall at our institution and was similar to women’s representation among faculty nationally. Departments with high percentages of women faculty (Obstetrics and Gynecology, 76%; Pediatrics, 65%) had high percentages of women GR speakers (Obstetrics and Gynecology, 70%; Pediatrics, 51%; Psychiatry, 62%). COVID-19 did not appear to significantly influence gender representation among speakers.

Conclusion

At our institution, less than half of the GR speakers were women. However, this percentage appears to reflect the overall percentage of women faculty. Potential barriers and opportunities resulting from the COVID-19 pandemic did not appear to impact this finding.

## Introduction

The number of women enrolled in medical schools across North America currently exceeds 50% [1]. Yet, there are fewer women the higher one goes in academic rank and organizational leadership within academic medicine [2]. Grand rounds (GR) are formal opportunities for sharing professional expertise. An invitation to speak at GR is considered an honor in our profession and a critical activity for academic promotion. Prior studies have described gender disparities in the number of presentations and speaking time allotted at GR and international conferences [3-5]. Such inequities have the potential to impact women’s professional advancement and may limit role-modeling and networking opportunities for women trainees.

Amidst the COVID-19 pandemic, there has been a global socio-economic upheaval that has highlighted and exacerbated existing gender disparities in medicine [6-10]. We aimed to study women’s representation among GR presenters at an academic health system in Western Massachusetts before and during the pandemic. We hypothesized that women would be less represented as speakers compared with men and that this representation would worsen following the COVID-19 pandemic given increasing child and family care responsibilities [6,11,12].

## Materials and methods

We conducted an exploratory, single-center retrospective cross-sectional study. In this study, we describe gender differences in GR speakers at Baystate Health (BH), a large, diversified health system that is the primary clinical affiliate of University of Massachusetts Chan School of Medicine’s regional campus located in Springfield, Massachusetts. The institution has 11 Accreditation Council for Graduate Medical Education (ACGME)-approved residencies and 18 fellowships. We assessed all GR speakers (physician and non-physician) from January 2019 to June 2021 across nine academic departments: Anesthesiology, Emergency Medicine, Internal Medicine, Obstetrics and Gynecology (OBGyn), Pathology, Pediatrics, Psychiatry, Radiology, and Surgery. Due to the low frequency of GR in Pathology and lack of a formal GR structure in Neurology, we were unable to generate stable estimates of gender representation for this department.

We identified GR speakers from departmental administrators and cross-checked this data with publicly available information on EthosCE, BH’s learning management system for Continuing Medical Education (CME) during our study period. To our knowledge, all departmental GR committees invited speakers without any standard guidelines. Authors inferred speaker gender identification via internet searches and information available on the websites of speakers’ home institutions. We did not contact speakers or their primary institutions directly to confirm this gender we assigned. We excluded speakers if records did not note a speaker’s name. Additionally, we obtained the percentage of faculty members by gender in each BH department from the Office of Faculty Affairs at our regional medical school campus (UMass Chan Medical School - Baystate). We utilized 2021 data from the Association of American Medical Colleges to identify the national-level percentages of women faculty by department [13].

On March 13, 2020, the United States Government declared COVID-19 as a national emergency requiring shutdown of all offices and limiting in-person contact. Hence, we considered all GR delivered prior to that day as pre-COVID presentations. Since GR are scheduled in advance, using the date of March 13, 2020, as the cut-off assumes that the pandemic declaration could have differentially impacted the immediate cancellation or rescheduling of men or women presenters versus planned scheduling decisions. Thus, we repeated our analysis allowing for approximately a three-month lag (June 15, 2020).

We performed a descriptive analysis using frequencies, percentages, and absolute differences to illustrate how women were represented in GR at our institution overall and at the department level and how the COVID-19 pandemic may have influenced the representation overall. We estimated 95% confidence intervals for absolute differences to represent the variability of our estimates. Analysis was conducted using Stata/MP v 17.0 (StataCorp, LLC, College Station, TX). This study received an exemption from the Institutional Review Board at BH.

## Results

Pre-pandemic, there were 607 speakers across all departments: 276 (46%) were women, which was slightly higher than the overall representation of women faculty across the hospital system and similar to the percentage of women nationally (Table 1). Within departments, the percentage of women speakers ranged from 32% to 70%. Departments with the highest percentage of women faculty at BH (OBGyn, 76%; Pediatrics, 65%) had a high percentage of women speakers (70% and 51%, respectively). However, Psychiatry had a higher number of women speakers (62%) despite having fewer women faculty (46%). Although Surgery and Anesthesia had the lowest percentage of women faculty at BH (Surgery, 15%; Anesthesiology, 19%), women represented 41% and 32% of their GR speakers, respectively.

**Table 1:** Distribution of women grand rounds presenters at Baystate Health (Jan 2019-June 2021) and the percentage of women physicians at our institution (2021) and nationally (2021) ^a^'Women faculty locally' implies the percentage of women faculty at Baystate Health. ^b^Percentage of women faculty nationally as of December 2021 obtained from https://www.aamc.org/data-reports/faculty-institutions/interactive-data/2021-us-medical-school-faculty

	Total	Men speakers	Women speakers	Women faculty locally^a^	Women faculty nationally^b^
Overall	607	331 (55%)	276 (46%)	42%	44%
Department					
Anesthesiology	122	83 (68%)	39 (32%)	19%	37%
Emergency Medicine	29	16 (55%)	13 (45%)	41%	39%
Internal Medicine	125	73 (58%)	52 (42%)	38%	42%
Obstetrics/Gynecology	46	14 (30%)	32 (70%)	76%	68%
Pathology	3	2 (-)	1 (-)	42%	44%
Pediatrics	152	74 (49%)	78 (51%)	65%	61%
Psychiatry	34	13 (38%)	21 (62%)	46%	56%
Radiology	27	15 (56%)	12 (44%)	35%	30%
Surgery	69	41 (59%)	28 (41%)	15%	28%
Speaker affiliation					
External	209	118 (57%)	91 (44%)	-	-
Internal	398	213 (54%)	185 (47%)	-	-

In terms of the impact of COVID-19 at our institution, we did not observe meaningful differences in the overall percentage of women GR speakers pre-pandemic (45%) versus during the pandemic (46%) when using the March 2020 cut-off (absolute difference = 0.8%; 95% confidence interval -7% to 9%). When allowing for a three-month lag (June cut-off), we continued to observe minimal differences pre-pandemic (46%) versus during the pandemic (45%) (absolute difference = -1.7%; 95% confidence interval -10% to 6%).

## Discussion

We observed that less than half of GR speakers at our institution were women during the study period, albeit with a smaller gender disparity among speakers than observed in studies conducted elsewhere [3,5,14]. Moreover, based on the percentage of women among faculty locally and nationally, women speakers were either appropriately represented or over-represented in most departments. While we did not explore the reason for this finding in our current investigation, we hypothesize that it could be attributed to a well-publicized research effort within our Department of Surgery that preceded our study period. Faculty colleagues in Surgery previously examined the prevalence of sexual harassment within their department, published their findings, and presented their results across BH via a series of GR talks [15]. During these presentations, study authors highlighted the need for promoting women faculty well-being. As a result, GR planning committees may have been more intentional about inviting women GR speakers. Higher proportions of women GR speakers compared to women faculty in our Departments of Surgery and Anesthesiology may also reflect recent efforts within the surgical community nationally to promote gender equity [16]. We observed that the COVID-19 pandemic did not result in an overall decrease (or increase) in representation of women as GR speakers at our institution, which is heartening amidst the pandemic’s deleterious effects on women in our profession observed nationally [6,9].

Limitations of our investigation include the experience of a single institution that may not be generalizable. In addition, we did not assess how our departments recruited GR speakers or the number of GR invitations extended but declined. These elements should be considered for future study. Furthermore, any changes to these recruiting practices before and during the pandemic could have impacted our findings. However, to our knowledge, no such alterations occurred. Finally, we made the assumption that there were no major changes in the percentages of women faculty locally or nationally over the course of the study period; however, given the “great resignation”, this assumption may not be reasonable [17].

## Conclusions

At our institution, less than half of GR speakers were women; however, this percentage approximates the percentage of our women faculty overall. The restrictions of the COVID-19 pandemic did not appear to modify the representation of women as GR speakers.

## References

[1] Jena AB, Khullar D, Ho O, Olenski AR, Blumenthal DM (2015). Sex differences in academic rank in US medical schools in 2014. JAMA.

[2] Richter KP, Clark L, Wick JA (2020). Women physicians and promotion in academic medicine. N Engl J Med.

[3] Buell D, Hemmelgarn BR, Straus SE (2018). Proportion of women presenters at medical grand rounds at major academic centres in Canada: a retrospective observational study. BMJ Open.

[4] Mortaji P, Barrett E (2023). Gender differences in internal medicine grand rounds - speakers at a local academic hospital. https://digitalrepository.unm.edu/hsc_ed_day/9.

[5] Sharpe EE, Moeschler SM, O'Brien EK, Oxentenko AS, Hayes SN (2020). Representation of women among invited speakers for grand rounds. J Womens Health (Larchmt).

[6] Woitowich NC, Jain S, Arora VM, Joffe H (2021). COVID-19 threatens progress toward gender equity within academic medicine. Acad Med.

[7] Misra V, Safi F, Brewerton KA (2021). Gender disparity between authors in leading medical journals during the COVID-19 pandemic: a cross-sectional review. BMJ Open.

[8] Wenham C, Smith J, Morgan R (2020). COVID-19: the gendered impacts of the outbreak. Lancet.

[9] Brubaker L (2020). Women physicians and the COVID-19 pandemic. JAMA.

[10] Matulevicius SA, Kho KA, Reisch J, Yin H (2021). Academic medicine faculty perceptions of work-life balance before and since the COVID-19 pandemic. JAMA Netw Open.

[11] Alon T, Doepke M, Olmstead-Rumsey J, Tertilt M (2020). The Impact of COVID-19 on Gender Equality.

[12] Gewin V (2020). The career cost of COVID-19 to female researchers, and how science should respond. Nature.

[13] (2023). Active physicians by sex and specialty, 2019. https://www.aamc.org/data-reports/workforce/interactive-data/active-physicians-sex-and-specialty-2019.

[14] Boiko JR, Anderson AJ, Gordon RA (2017). Representation of women among academic grand rounds speakers. JAMA Intern Med.

[15] Wu JJ, Kartiko S, Kapil AM (2018). Surgery’s #MeToo movement: results of a sexual harassment survey in an academic institution. J Am Coll Surg.

[16] Rakestraw SL, Chen H, Corey B, Sutzko DC (2022). Closing the gap: increasing female representation in surgical leadership. Am J Surg.

[17] (2023). What is 'The Great Resignation'? An expert explains. https://www.weforum.org/agenda/2021/11/what-is-the-great-resignation-and-what-can-we-learn-from-it/.

